# Multi-parameter time series dataset recorded during blowdown of CO_2_ and mixtures from à 2m³ sphere, up to 130 bar and down to -50°C

**DOI:** 10.1016/j.dib.2025.112379

**Published:** 2025-12-15

**Authors:** Didier Jamois, Christophe Proust, Leila Teberikler, Adil Fahmi

**Affiliations:** a Ineris, Parc Technologique Alata, BP2, Verneuil-en-Halatte 60550, France; b Sorbonne University, TIMR Laboratory, UTC-ESCOM, Compiègne 60200, France; c TotalEnergies, EP Norge AS, Finnestadveien 44, Stavanger 4029, Norway; d Equinor, Arkitekt Ebbells veg 10, Trondheim 7053, Norway

**Keywords:** Carbon Capture Storage, CO_2_ vessel, Depressurization, Mass flowrate, Dry ice, Triple point state, Heat exchange rate

## Abstract

This dataset supports research on CO₂ blowdown phenomena relevant to the design of CCS/CCUS systems. Blowdown involves rapid depressurisation, causing complex heat transfer between gas and vessel walls. Modelling these processes is challenging, and comprehensive experimental data are limited, particularly those obtained at realistic scales.

Experiments were conducted using the TRIPHASYX research Infrastructure. This setup features pressure, temperature, and thermal flux sensors, as well as load cells to measure mass flow rate accurately. Tests involved pure CO₂, methane, and mixtures of CO₂ and CH₄ under various initial conditions.

The dataset comprises 10 experimental batches, each with seven synchronised Excel files containing time-series data. It captures temperature, pressure, heat flux, and mass flow rate during blowdown events. These large-scale experiments provide valuable insights for validating CO₂-specific models and enhancing the design of CCS/CCUS equipment.

Specifications Table

**Table:** 

Subject	Earth & Environmental Sciences
Specific subject area	Carbon Capture Utilisation and Storage (CCUS). Safety of CO_2_ storage and transport
Type of data	Time series, Excel format
Data collection	This dataset was achieved in 2019 during a test campaign using the Eric Eccsel (Home | ECCSEL) Research Infrastructure “Triphasyx”. This infrastructure is described in [1]. Data are essentially constituted of pressure, temperature, flux and mass measurements. These data were recorded during blowdowns from a 2 m³ vessel under various initial conditions of pressure and temperature.
Data source location	INERIS, France, Verneuil en Halatte
Data accessibility	Repository name: zenodo Data identification number: 10.5281/zenodo.17257910 Direct URL to data: https://doi.org/10.5281/zenodo.17257910
Related research article	D. Jamois, C. Proust, L. Teberikler, A. Fahmi, An experimental setup for investigating the blowdown of liquid CO2 down to -50°C, Int. J. Greenhouse Gas Control, 129 (2023) 103974, DOI: 10.1016/j.ijggc.2023.103974

## 1 Value of the Data

• Understanding heat transfer and thermodynamic phenomena during blowdown is essential for properly sizing CCS/CCUS equipment. The transient heat exchange between rapidly depressurising gas and vessel walls is a complex issue. Modelling these processes is challenging, and comprehensive experimental data are limited, particularly those obtained at realistic scales [[2], [3], [4], [5]].
• Existing simulation tools have been developed and calibrated using experimental data on hydrocarbons (methane, propane, mainly). However, due to significant differences in thermodynamic and physical properties between hydrocarbons and CO_2_—particularly the potential for phase transitions—these models must be validated with specific CO_2_ blowdown experiments.
• The dataset described here was obtained using experimental hardware described in [1], known as TRIPHASYX. The core of this setup is a 2 m^3^ sphere, which was used to produce CO_2_ blowdown starting from different initial conditions. Key parameters are measured during these blowdowns, including temperatures, pressures, thermal flux, and mass flow rate.
• The size of the vessel enables the production of unbalanced thermodynamic conditions that are difficult to reproduce at lab scale and are representative of what may occur during specific CCS operations, such as the loading and offloading of large tanks.
• Researchers and industry stakeholders involved in the design and sizing of CCS facilities will find in this dataset a valuable resource for validating and benchmarking their computational tools.

## 2 Background

Among the challenges identified in the deployment of Carbon Capture and Storage (CCS) at an industrial scale, the rapid blowdown of CO_2_ vessels, whether accidental or intentional, is a significant one. Existing simulation tools need to be benchmarked against dedicated CO_2_ blowdown experiments before they can be used confidently for CO_2_ mixtures. In this frame, INERIS offered its expertise from the experimental investigation of the blowdown of pressurised containers. A spherical 2m^3^ sphere, thermally insulated and connected to release pipes with discharge orifices to control the depressurisation rate, was used.

For CARDICE project, 10 blowdown experiments were performed with various initial conditions. The setup and its capabilities were described in [1]. The results obtained are now available in the form of a dataset presented here (Table 1).

**Table 1:** Initial conditions for the 10 experiments

Batch n°	Pressure (bar)	Mean temperature (°C)	Mass CH_4_ (kg)	Mass CO_2_ (kg)	Release diameter (mm)	Type of release	Ambient temperature (°C)
1	100	20	155		6	Gas	11
2	131	27	170*	126	6	Gas	14
3	130	27	132*	376	6	Gas	22
4	101	22	98*	270	6	Gas	20
5	21	-19		822	3	Gas	4-14
6	20	-19		802	3	Liquid	12-16
7	15	-27		791	4	Gas	9-10
8	15	-27		757	4	Liquid	13-15
9	10	-38		904	4	Gas	12-13
10	10	-37		743	4	Liquid	13-15

*The mass proportion of methane and CO_2_ represents a mixture of 20% CO_2_ / 80% CH_4_ molar for test n°2 and 50% CO_2_/50% CH_4_ molar for tests 3 and 4.

## 3 Data Description

The dataset is intended for research and analysis in the field of CCS/CCUS. It contains the measurements recorded during tests of fluid blowdown from a 2m^3^ reservoir under various initial conditions of pressure and temperature. This dataset includes 10 batches of experimental data, each representing an independent experimental session. Initial conditions for each batch are reported in the following table.

An experiment begins when the targeted initial conditions are reached. These initial conditions are recorded several tens of seconds before the opening of the release orifice.

Each batch includes seven Excel files (.xlsx), each containing time series data for a specific set of measured parameters. These files are named as indicated in Table 2 (with the batch index x). The column label for each type of file is specified.

**Table 2:** File identification

File name	Column label
Ex-pressures-1Hz-DP.xlsx	Time, Psphere, Prelease1, Prelease2
Ex-mass-flowrate-1Hz-DP.xlsx	Time, mass, flow rate
Ex-internal-temperatures-1Hz-DP.xlsx	Time,Tcin1,Tcin2,Tcin3,Tcin4,Tcin5,Tcin6
Ex-inside-wall-temperatures-1Hz-DP.xlsx	Time,Tc1,Tc2,Tc3,Tc4,Tc5,Tc6,Tc7
Ex-outside-temperatures-1Hz-DP.xlsx	Time,TcF1,TcF2,TcF3,TcF4,TcF5,TcF6,TcF7
Ex-pipe-temperatures-1Hz-DP.xlsx	Time,Tcrelease1,Tcrelase2
Ex-flux-1Hz-DP.xlsx	Time,Flux1,Flux2,Flux3,Flux4,Flux5,Flux6,Flux7

The timelines of the seven files belonging to a batch are synchronised. The raw data were initially recorded at a frequency of 250Hz; then, a low-pass filter (a running average over 250 samples) was applied to these data to reduce the file size to a rate of 1Hz.

The sensor types used to obtain these data are presented in Table 3.

**Table 3:** Types of sensors used

Parameter	sensors	uncertainty
Temperature	Thermocouple K – class 1	+/-0.4°C
Pressure	Kistler piezoresistive sensor (4043A)	+/- 0.2 bar
Mass	4 load cells 0-2000kg – Mettler Toledo – 0745A serie	+/-0.3 kg
Flux	Thermal microfluxmeter – Captec (www.captec.fr)	+/-1 W/m^2^

## 4 Experimental Design, Materials and Methods

The experimental setup and its functioning are detailed in [1]. The vessel is a 2 m³ steel sphere with an inner diameter of 1580 mm and a mean wall thickness of 60 mm. The total weight is about 4 tonnes. The vessel is designed to withstand a static internal pressure of 200 bar. It has three flanges with four ports for measurements, fluid introduction, and vessel content release. The sphere is insulated with a thermal conductivity coefficient of approximately 4.3 W/mK.

### 4.1 Pressure, temperature and flux measurements

Three pressure transducers are used simultaneously to measure the pressure inside the sphere and in the pipes (for gas and liquid release), upstream of the release orifice. Note that the pressure measurement in the pipe is associated with a temperature measurement at the same point, offering the possibility of estimating local thermodynamic parameters, such as density, using pressure and temperature as inputs into a suitable equation of state.

**Fig. 1:**
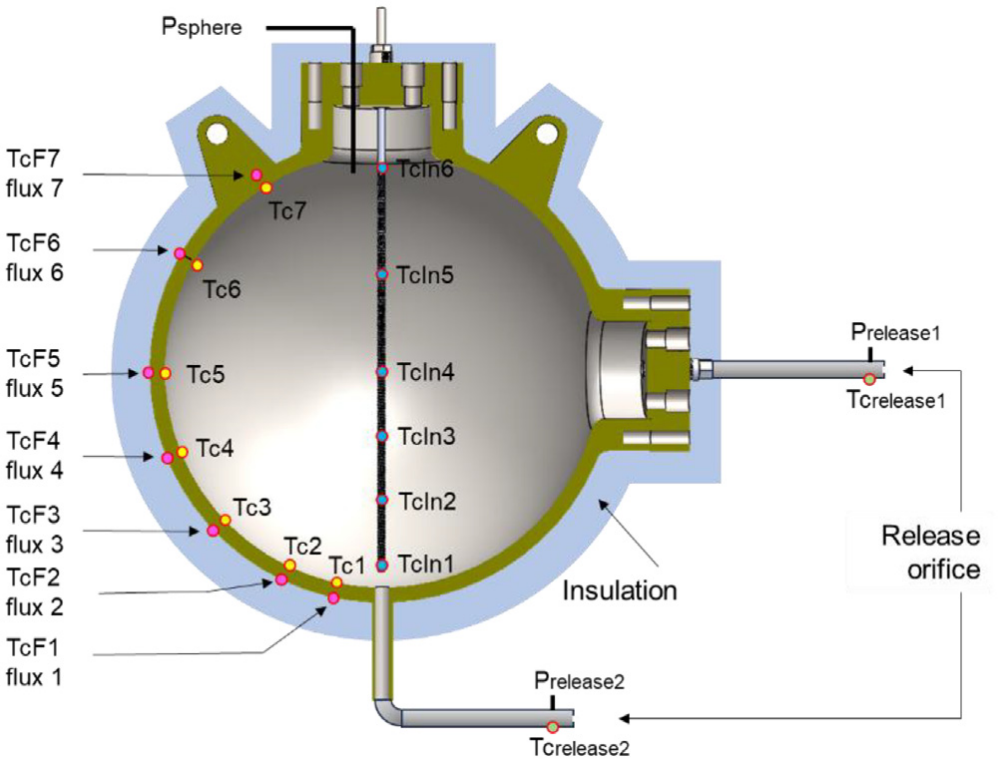
schematic of the 2m^3^ sphere with sensor location and their label

The fluid's temperature is measured using thermocouples at 6 locations inside the vessel. The thermocouples are fixed on a vertical rod at the positions indicated in Fig. 1.

Seven thermocouples are inserted into tiny holes drilled from the outside surface (Fig. 1), with the tips ending 5 mm from the inner surface. This way, the local (inner wall) temperature is obtained with limited contact bias. Seven temperature measurements are placed at the same location, but on the outside surface. These last thermocouples are associated with seven microfluxmeters, which are glued to the surface of the sphere. These microfluxmeters provide voltage outputs directly proportional to the flux. The uncertainty mentioned in Table 3 was obtained from the ratio signal/noise measured in steady state conditions.

The exact location of each of these sensors (P, T, Flux) is indicated in [1].

### 4.2 Mass flow rate measurements

The sphere is weighed using four load cells. The mass flow rate is the derivative of the mass versus time curve. Since a monotonous mass loss is expected during a release, the mass curve should be a linear function of time over short enough periods, typically several seconds. Once such a “short enough period of time” is defined, a linear regression can be applied to obtain the slope, which provides the local mass flow rate.

The standard error of the slope (often denoted as SE_β1_) measures the accuracy with which the regression slope (β1) estimates the exact relationship between time and mass.

It is calculated using the following:

$$SE_{\beta 1}=\frac{s}{\sqrt{\sum {\left(t_{i}-\bar{t}\right)}^{2}}}$$

Here, s is the standard error of the estimate:

$$s=\sqrt{\frac{1}{n-2}\sum {\left(m_{i}-{\hat{m}}_{i}\right)}^{2}}$$

With ${\hat{m}}_{i}$, the ieme term of the linear regression.

Here, n represents the number of samples taken during the time over which the slope is calculated. It is linked to the data logger's recording rate.

The mass flow rate uncertainty may be estimated using a time serie representing a mass decrease with a random scattering of ±0.3 kg applied to each individual mass point (the mass uncertainty reported in Table 3).

This simulation indicates that a minimum of 150 samples is required to achieve a relative uncertainty of less than ±1%. In practice, the recording rate is set to 250Hz, which is the load cell conditioner's cut-off frequency. A higher recording rate does not further improve the uncertainty because the conditioner provides constant values during the interval between two frequency steps.

### 4.3 Mixtures production

The sphere can be directly pressurised with methane using gas bundles. A commercial cryogenic reservoir is used to fill the sphere with pure CO_2_ at a temperature of -20°C. Once introduced, the fluid can be warmed or cooled.

It was intended to produce monophasic methane/CO_2_ mixtures at ambient temperature and pressure up to 130 bar. The required quantity of liquid CO_2_ is first introduced into the sphere, and the fluid is heated above its critical point (up to 35°C). Methane is then rapidly injected in the dense phase (so that an intense jet and swirls are produced). Two sampling lines, one in the upper part of the sphere and another at half-height in the release pipe, control the final homogeneity of the mixture. Catharometric gas sensors (based on thermal conductivity) measure CH_4_ concentration in CO_2_. They achieve an accuracy of approximately +/-0.5 % v/v CH_4_. Once the mixture is obtained, it takes two days to cool the sphere and its contents to ambient temperature.

Using evaporation cooling, pure liquid CO_2_ may be brought to almost -50°C. This method is used to obtain initial conditions for pure CO_2_ release tests. However, the temperature gradient of the sphere is initially significant, and it may take several days to reach a nearly equilibrium state.

## Limitations

As pointed out previously, the size of the vessel allows to observe unbalanced thermodynamic situations. The main drawback is that initial conditions (temperatures in the gas phase mainly) may appear heterogeneous and unrepeatable. These conditions result from former operations (previous test, filling of the sphere, standby period, outside conditions) which affect temperature gradients in the gas phase and in the sphere wall, even if time is allowed to reach a steady state before a release.

As mentioned, temperatures, pressures, flux and mass were recorded at a rate of 250Hz and then processed with a running average method over 250 samples to obtain 1Hz timeline reduced files. This method provides filtered and low size files. The mass flow rate was calculated from the 250 Hz mass raw data with the method described previously (running slope over 250 samples) and then reduced to 1Hz, synchronized with the other files.

For the experiments that lasted several hours, a data buffering issue necessitated interrupting the recording for a few minutes during the test. These interruptions are visible on the corresponding time curve for batches 5, 7, 9 and 10.

## Ethics Statement

This manuscript adheres to the Ethics in Publishing standards.

## CRediT Author Statement

**Didier Jamois**: investigation, data curation, methodology, resources, writing – Original Draft, writing – review & editing; **Christophe Proust**: validation, formal analysis, investigation, supervision; **Leila Teberikler**: conceptualisation, supervision, software; **Adil Fahmi**: conceptualisation, supervision, software.

## Data Availability

zenodoAccess status 1 Resource types 1 Subjects 1 1 1 1 1 1 1 File type 1 Help Search guide October 7, 2025 (v1)DatasetOpen Multi-parameter time series dataset recorded during blowdown of CO2 and (Original data).
